# Supplementary material for: Effect of Aromatic Herbs and Spices Present in the Mediterranean Diet on the Glycemic Profile in Type 2 Diabetes Subjects: A Systematic Review and Meta-Analysis
Source: Nutrients. 2024 Mar 7;16(6):756. doi: 10.3390/nu16060756 (PMC10975382; doi:10.3390/nu16060756)
Supplement: Supplementary file 1 [file nutrients-16-00756-s001.zip › Supplemental Tables S2 and S3.pdf]

Supplemental Table 2. Articles included in the systematic review which do not meet the inclusion criteria to be included in the meta-analysis study.

First author, year of publication	N	Participants	Male, n (%)	Age, years	Study design	Intervention dose mg/day (number of subjects)	Duration (days)	Body weight (kg)		Glucose (mg/dL)		Hb1Ac, (%)		Insulin (UI/μL)		Quality checklist mean
								Baseline	Final	Baseline	Final	Baseline	Final	Baseline	Final	
**Moghadam *et al.*, 2021**	60	T2DM obese men, 30–50 years, BMI > 30 kg/m^2, T2DM duration >2 years. Exclusion criteria were hepatic, renal, bone and CVD, severe hypertension, having chronic complications of diabetes and taking medications except for oral hypoglycaemic agents.	60 (100)	39 ± 5	Randomized, double-blind and parallel prospective clinical trial. Participants were randomly divided to four groups: training + placebo (CT; n = 15), saffron (S; n = 15), training + saffron (CTS; n = 15) or control (CON; n = 15). Training exercise include warm-up (5-10 min) and 1-1.5 h/day of HIIT with lifting	Group CT (n = 15) training + placebo: received one pill of 100 mg of maltodextrin daily.	84 days	93.9 ± 6	<0.001	NR	<0.001	NR	<0.001	NR	<0.001	0.695
						Group S (N=15) received one pill of 100 mg of pure saffron daily			<0.001	NR	<0.001	NR	<0.001	NR	<0.001	
						Group CT (n = 15) training + saffron: received one pill of 100 mg of saffron daily.			<0.001	NR	<0.001	NR	<0.001	NR	<0.001	
						Control group did not receive any pill and were asked to maintain a normal daily life pattern			NSD	NR	NSD	NR	NSD	NR	NSD	
**Assaad-Khalil *et al.*, 2022**	232	T2DM who have not received any previous antidiabetic medications. Exclusion criteria were pregnant or breast-feeding women, BMI >40 Kg/m^2 or < 18.5 Kg/m^2; eGFR <60 mL/min/1.73m^2, infection diseases, T1DM, or diabetic complications among others.	23 (35.9)	49 (16)	Double-blind, double-dummy, randomized, active-controlled, parallel-group, interventional, phase 2 clinical trial. Eligible patients were randomized group 1 who received metformin; group 2, who received low-dose NW Low-Glu® and group three, who received high-dose NW Low-Glu®.	Group 1: (N=64) received 2000 mg of metformin per day	84 days	91.2 (10.6)	90.4 (10.9) *	162.9 (62.6)	154.5 (68.5)	8.7 (1.9)	7.7 (1.6) *	NR	NR	0.803
			32 (49.2)	50 (14)		Group 2: (N=65): low-dose NW Low-Glu, which contained 1200 mg Mas Cotek, 400 mg Cinnamomum and 1000 mg black seed		88.4 (12.5)	87.5 (12.8) *	158.4 (72.7)	144.8 (53.6)	8.5 (1.4)	7.8 (1.6) *	NR	NR	
			32 (46.4)	39 (13.5)		Group 2: (N=65): high-dose NW Low-Glu., which contained 1500 mg Mas Cotek, 500 mg Cinnamomum and 1250 mg black seed		85.6 (12.5)	84.5 (12.2) *	169.5 (71.7)	159.2 (50.4)	8.8 (1.9)	8.1 (1.8) *	NR	NR	
**Quirarte-Baez *et al.*, 2019**	40	T2DM treatment with metformin and glibenclamide. 27.5% of them have dyslipidemia	13 (32.5)	56.3 ± 9.97	Intervention one arm, no-randomized and no-blind study	Each patient was provided with a box containing 30 sachets of rosemary leaves (2g). The patients must add a bag in litter of boiling water (boiled for 3 min). Patients must drink this water during the day	90 days	74.4 ±14.2	73.5 ± 14.7	181.2 ± 80.8	163.0 ± 72.9	9.09 ± 2.72	7.72 ± 1.82 *	2.29 ± 0.98 mUI/mL	1.12 ± 0.75 mUI/mL *	0.250
**Moustafa *et al.*, 2019**	44	T2DM subjects 18–60 years and T2DM diagnosed within a time duration ≤168 days. Other antidiabetic medications, pregnant and	7 (24.1)	NR	Prospective, open-label randomized clinical trial. Eligible patients were randomly in two groups: Group I: received metformin while Group II: received	Group I (N=23): received metformin tablets 2000 mg per day	84 days	87.7 ± 17.0	83.3 ± 15.4 *	166.2 ± 52.8	120.7 ± 25.4 *	7.58 ± 1.63	6.55 ± 0.72 *	12.7 ± 5.9	11.5 ± 5.3	0.696
			11 (30.6)	NR		Group II (N=21): received Black cumin oil capsules (Baraka) 450 mgs three times daily		86.9 ± 17.9	84.4 ± 17.1 *	142.5 ± 50.4	119.8 ± 23.7 *	7.44 ± 1.16	7.01 ± 0.83	18.2 ± 12.9	16.5 ± 13.1	

		lactating women among others were excluded.			Black cumin oil capsules three times daily.											
**Bamosa *et al.*, 2010**	94	T2DM uncontrolled diabetes, with several Hb1Ac>7%, age 18–60 years, treated only with oral hypoglycemic drugs. Patients with chronic cardiac, hepatic and renal illness	14 (46.7)	47.80± 1.42	Intervention study. All patients were divided in three groups, which received 3 different oral doses of Black cumin (1 g, 2 g, and 3 g per day)	Group 1: received 1g of Black cumin per day	84 days	33.6 ± 1.53 kg/m^{2†}	33.3 ± 1.53 kg/m^{2†}	189 ± 14.3	171 ± 7.8	8.36 ± 0.31	8.01 ± 0.27	NR	NR	0.452
			14 (43.8)	49.63± 0.97		Group 2: received 2 g of Black cumin per day		28.9 ± 0.95 kg/m^{2†}	29.4 ± 0.94 kg/m^{2†}	219 ± 12.3	162 ± 9.2*	9.09 ± 0.24	7.57 ± 0.30*	NR	NR	
			15 (46.9)	44.91± 1.88		Group 3: received 3g of Black cumin per day		31.63 ± 1.47 kg/m^{2†}	31.61 ± 1.50 kg/m^{2†}	204 ± 18.2	169 ± 16.4*	9.35 ± 0.41	7.31 ± 0.37*	NR	NR	
**El-Shamy *et al.*, 2011**	66	This study included 41 T2DM subjects without diabetic complication and 25 healthy non-diabetic subjects	NR	NR	Intervention study. T2DM subjects received Black cumin plus their oral antidiabetic drug, while healthy subjects only received Black cumin.	T2DM group (N=41): 2.5 grams of N. sativa + their oral antidiabetic drug per day	168 days	NR	NR	148.7 ± 26.59	127.6 7 ± 22.01*	7.18 ± 0.83	6.02 ± 0.58*	NR	NR	0.372
			NR	NR		Healthy group (N=25): 2.5 grams of N. sativa per day		NR	NR	80.22 ± 10.8	73.34 ± 8.71*	4.43 ± 0.36	4.14 ± 0.47*	NR	NR	
**Bilal *et al.*, 2019**	75	T2DM subjects, FGB more than normal values, age 30-60 years, not insulin therapy and not another chronic disease.	31 (41.5)	47.80 ± 1.10	Intervention study one arm. T2DM subjects received Black cumin treatment for first 40 days and placebo treatment for another 40 days. Only 41 subjects completed the study	Black cumin treatment included 0.7 g of seeds of Black cumin per day.	80 days (40 days with N. sativa and 40 days with placebo)	NR	NR	190.780 ± 8.042	168.317 ± 7.150* after N. sativa	NR	NR	186.487 ±7.491	13.194 ±1 .404* after N. sativa	0.464
											186.487±7 .491 after placebo				8.850 ± 0.694* after placebo	
**Ali *et al.*,**	60	T2D subjects with HbA1c ≥ 7 % and ≤ 7.5 %, aged 18- 65, BMI 18- 30 kg/m^2, and stable dose of metformin (1000 mg/day) for the last 84 days.	9 (69.2)	50 ± 3.1	A randomized, open label, prospective, three-arm, parallel, multicenter study. All T2DM subjects were randomized in 3 arms: Test arm 1 (T1), Test arm 2 (T2), and reference arm 3 (R). Out of 60 patients, 45 patients completed the study (T1 (n = 13), T2 (n = 18) and R (n = 14)	Test arm 1 (N=13): received 1 tablet of metformin 1000 mg once daily and 1 tablet of Thymoquinone 50 mg	90 days	63.3 ± 3.1	NR	144.0 ± 21.6	114.3 ± 8.6	7.2 (0.1)	6.7 (0.1)	NR	NR	0.416
			8 (44.4)	48 ± 2.5		Test arm 2 (N=18): received 1 tablet of metformin 1000 mg and 2 tablets of Thymoquinone 50 mg		65.8 ± 2.3	NR	118.0 ± 5.6	103.2 ± 4.4	7.2 (0.1)	6.8 (0.1)	NR	NR	
			9 (64.3)	49 ± 1.7		Test arm 3 (N=14): received only 1 tablet of metformin 1000 mg		67.0 ± 2.4	NR	129.7 ± 6.9	111.4 ± 6.3	7.3 (0.1)	7.1 (0.1)	NR	NR	
**Zarvandi *et al.*, 2017**	30	T2DM subjects with T2DM diagnosed at least for 2 years duration, > 25 years, LDL>100mg/dL, TG>150 mg/dL and taking statins and hypoglycemic drugs. T1DM, pregnancy, insulin therapy, renal	11 (36.7)	56 ± 8	The open-label Phase I clinical trial. The selected patients were orally administered one sachet two times daily (before a meal) for 40 consecutive days.	Each PHF sachet (6.4 g) consisted of a combination of 300 mg A. sativum, 300 mg A. vera, 1.8 g N. sativa, 1 g P. psyllium, 2.5 g T. foenum-graecum, and 500 mg S. marianum extract. All subjects also continuously received their statins and oral hypoglycemic	40 days	78 ± 12	77 ± 12	162 ± 40	146 ± 37*	8.4 ± 1.5	7.7 ± 1.1*	NR	NR	0.393

		o liver disease were excluded				agents.										
**Rao *et al.*, 2020**	40	15 Obese; 9 T2DM; 16 T2DM with obesity. Obese was defined as BMI>25 kg/m². T2DM was defined as FBS >110 mg/dL and/or Hb1Ac>6%	5 (33.3)	37.0 (32–42)	Exploratory, interventional, single-arm trial. Results were obtained depending on their clinical characteristics: Obese, T2D or T2D + obesity	All patients received a wheat flour chapatis, fortified to provide, respectively, 4.7 and 0.75 g powdered Black cumin and fenugreek seed/day	84 days	74.1 ± 1.98	Week change -2.12 (-3.13 to -1.10)	92.9 ± 1.99	Week change -6.95 (-15.0 to 1.05)	5.29 ± 0.0661	Week change -5.65 (-8.59 to -2.85) *	NR	NR	0.738
			0 (0)	54.4 (42–63)				69.4 ± 1.88		137 ± 15.1		7.92 ± 0.398		NR	NR	
			5 (32.3)	54.2 (41–72)				74.5 ± 3.22		164 ± 11.3		7.97 ± 0.392		NR	NR	
**Banerji *et al.*, 2016**	50	T2DM not insulin dependent, age 30-60 years, HbA1c: 7.5% - 10%. Patients with chronic disease, alcohol drinkers and pregnant/lactating women were excluded	20 (40)	50.62 (7.48)	Prospective, single arm, open label investigation trial. Of total recruited patients, only 35 completed the study	Capsules contained 350 mg total extracts of fenugreek (Trigonella foenum-graecum, 110 mg), Indian gooseberry (Emblica officinalis, 90 mg), turmeric (Curcuma longa, 70 mg) and grape seed (Vitis vinifera, 80 mg).	84 days	27.14 (4.15) kg/m²†	26.59 (3.98) kg/m²†	9.19 (1.92) mmol/L	8.15 (2.78) mmol/L	8.70 (0.87)	8.13 (1.39) *	NR	NR	0.378
**Faizal *et al.*, 2009**	43	T2DM subjects	NR	35-75	Intervention study where patients were divided into 6 groups based on their age group and also according to the mean FBS values. As control group normal healthy subjects were taken. All groups received a tablet which contains: Salacia oblonga wall-250 mg, Curcuma longa L-125 mg, Emblica officinalis-125 mg	Group I -Normal Control Subjects (n=10)	84 days	NR	NR	87.521 ± 9.44	NR	5.50 ± 0.22	NR	22.72 ± 6.19		0.265
			NR	35-45		Group II - 35-45 years (n=15)		NR	NR	168.19 ± 56.55	142.10 ± 41.74 *	8.3 ± 3.7	6.71 ± 2.17*	18.3 ± 6.88	26.27 ± 5.94*	
			NR	46-55		Group III - 46-55 years (n=13)		NR	NR	148.82± 34.61	124.53±3. 34	7.754±2.9 3	5.875±1.2 6	15.53±6 .03	23.72±5 .41	
			NR	>55		Group IV - > 55 years (n=15)		NR	NR	148.01± 36.03	122.953±27 .16*	7.267±2.6 4	6.239±1.2 1	16.333± 6.67	24.227± 6.35*	
			NR	NR		Group V - FBS < 145.9 mg/dl (n=21)		NR	NR	121.39± 9.78*	104.40±15 .53	5.83±1.06	5.39±0.52 *	17.71±7 .10	25.08±6 .38*	
			NR	NR		Group VI - FBS > 145.9 mg/dl (n=22)		NR	NR	171.45± 20.58	141.87±23 .11*	9.53±3.68	7.03±2.05 *	15.541± 6.08	24.50±5 .94*	
**Ferreira de Sousa *et al.*,2020**	89	T2DM subjects not-insulin therapy, age>18 years. Patients with immunosuppression and chronic disease were excluded	5 (17.9)	NR	Participants were randomized in three groups: one supplemented with turmeric and piperine; other supplemented with passion fruit albedo and another one with control group.	Group CURPI (N=33): received capsules of long turmeric (500 mg) and piperine 5mg once a day, 60 minutes before lunch	112 days	74.07 ± 13.88	73.98 ± 13.74	203.97 ± 103.64	197.33 ± 118.53*	8.64 ± 2.99	8.57 ± 2.90*	12.24 ± 11.16	12.18 ± 7.90	0.768
			10 (30.3)	NR		Group FAMA (N=28): received 500 mg of flour obtained from the rind of the yellow passion fruit		75.02 ± 17.9	73.8 ± 13.8	172.93 ± 83.73	162.68 ± 74.88*	7.91 ± 2.37	7.84 ± 2.31*	12.80 ± 10.59	12.93 ± 8.11	
			4 (14.3)	NR		Control group (N=28): did not receive any supplementation		67.98 ± 12.4	67.98 ± 11.2	138.75 ± 77.01	151.29 ± 62.95*	7.25 ± 1.60	7.21 ± 1.43*	10.50 ± 8.56	10.04 ± 6.94	
**Iyer *et al.*, 2010**	55	Stable T2DM patients	12	52±6	Intervention study with 3-arms. Patients were divided in three groups:	Control group (N=20): no / Subjects with FBS>150	90 days	71.17 ± 5.98	NR	199.60± 40.22	189.85±54 .45*	9.46±0.90	9.10±1.08	NR	NR	0.480

					control, fresh and processed group. Both fresh and processed supplementation was a mix of herbs, which included amla, tulsi, mint, ginger and turmeric. Results was reported depending on their FBS values	supplementation.	Subjects with FBS<150				115.31±17.55	125.51±18.08	6.63±1.76	7.32±0.59	NR	NR	
			11	61±7		Fresh group (N = 15) 150ml of fresh panchratna (30g pulp) juice daily	Subjects with FBS>150	45 days	69.18 ± 4.73	NR	201.48±38.5	183.03±50.2	8.92±1.18	8.40±1.36	NR	NR	
							Subjects with FBS<150				122.7±11.73	118±16.20	6.96±0.26	6.93±0.29	NR	NR	
			14	60±8		The processed group (N=20) bottled with 30g of panchratna pulp	Subjects with FBS>150	90 days	68.71 ± 8.02	NR	245.77±50.85	193.76±42.06	10.02±1.45	9.21±1.16	NR	NR	
							Subjects with FBS<150				125.67±12.77	121.25±17.24	7.35±0.72	7.13±0.41	NR	NR	
**Kurian *et al.*, 2014**	89	T2DM subjects with age 35-60 years, all patients must be under medical treatment, but showing FBS > 140 mg/dl. Patients with HbA1c>9.2%, chronic disease or pregnancy women were excluded.	50 (56.2)	35-60 years	Randomized case control study. All subjects were randomly assigned to receive treatment with G-400 and were asked to continue their usual treatment regimen for diabetes mellitus along with G-400.	Each patient was asked to take 100mg G-400		56 days	24.5 ± 3.9 $kg/m^{2\dagger}$ in male 25.7 ± 4.1 $kg/m^{2\dagger}$ in female	NR	184.84	127*	8.58	7.98*	NR	NR	0.378
**Mahajan *et al.*, 2015**	32	T2DM non-insulin dependent diabetes subjects, ages of 30-65 years, BMI 18.5 to 40. Patients with another chronic disease, high alcohol intake, smokers and T1D were excluded	27	55.59±1.47	Intervention study. All patients were asked to avoid a carbohydrate rich diet and advised regular walking for about 4-5 km during the course of study	"GSPF" consisted of a mixture of 10 herbs (Gymnema sylvestre, Syzygium cumini, Phyllanthus emblica, Curcuma longa, Pterocarpus marsupium, Terminalia chebula, Cassia fistula, Picrorhiza kurroa, Swertia chirata and Terminalia bellerica).10 g polyherbal powder was soaked in 50 ml of water and administered in an empty stomach		168 days	25.18 ± 0.70 $kg/m^{2\dagger}$	24.27 ± 0.72* $kg/m^{2\dagger}$	168.23 ± 5.35	128.70 ± 4.24*	7.21 ± 0.12	6.37 ± 0.09	NR	NR	0.467
**Mani *et al.*, 1997**	38	T2DM non-insulin dependent	15 (50)	54.00 ± 7.40	Intervention trial. All patients were divided in two groups: intervention and control group	Intervention group (N=30): received 80 mg of cereal mix, which included 0.3g Turmeric/100g		2 months	61.50 ± 5.70	NR	9.26 ± 2.89	6.54 ± 2.79*	NR	NR	NR	NR	0.273
			8 (100)	52.75 ± 3.14		Control group (N=8): did not receive supplementation			60.75 ± 1.54	NR	8.64 ± 0.70	8.58 ± 0.62	NR	NR	NR	NR	
**Singh *et al.*, 2016**	56	T2DM not-insulin dependent, age 30-70 years. Uncontrolled	NR	30-70 years	Conduct clinical trial study. All the diagnosed patients were divided	Group A (N=36): Chanaka Yoga (chickpea, curcumin, berberine, chebula, baheda,		90 days	NR	NR	157.74 ± 24.45	112.55 ± 12.38*	8.82 ± 1.20	7.32 ± 0.64*	NR	NR	0.308

		DM, another chronic disease and pregnancy women were excluded.			into two groups (Group A, Group B), Group A: Supplemented with a species mix named Chanaka Yoga group and Group B: Glimepiride treated	Indian gooseberry) 10 g 1 time a day before meal										
			NR			Group B (N=20): Glimepiride (1 mg) drug was given 2 times a day before meal		NR	NR	137.10 ± 10.67	114.60 ± 7.60	8.13 ± 0.96	7.68 ± 0.66*	NR	NR	
**Sukandar *et al.*, 2010**	32	T2DM subjects with dyslipidaemia, aged 35-75 years. Another chronic disease and women pregnancy were excluded	10 (90.9)	59.3 (6.83)	Double blind, randomized study. Patients were randomized in three groups: A, B or C. Patients have regular visit every two weeks	Group A (N=11): received 1.2 g of allium-curcuma	84 days	25.5 (3.26)	24.8 (3.21)	117.6 (52.1)	146 (52.7)	10.1 (3.04)	NR	NR	NR	0.462
			9 (90.0)	54.4 (4.36)		Group B (N=10): received 1.6 g of allium-curcuma		27.6 (4.83)	26.7 (4.52)	177.2 (45.0)	157 (34.4)	9.60 (1.69)	NR	NR	NR	
			8 (72.7)	54.5 (8.40)		Group C (N=11): received 2.4 g of allium-curcuma		29.2 (3.46)	27.9 (3.56)	175.1 (51.0)	139.4 (57.9)	11.48 (1.47)	NR	NR	NR	
**Sukandar *et al.*, 2014**	29	T2DM subjects, older than 35 years old and with or without dyslipidemia	NR	53.12 ± 2.11	Double blind, parallel, randomized control trial. All patients were divided in two groups: group AC received Allium Curcuma while group G received Glibenclamide	Group AC (N=17): received was 400 mg capsule contains 200 mg of turmericethanolic extract and 200 mg of garlic aqueous extract per day	14 weeks	25.80 ± 1.05 kg/m^{2†}	24.77 ± 1.00 kg/m^{2†}	192.76 ± 14.59	141.71 ± 9.67*	10.41 ± 0.64	8.09 ± 0.37*	56.89 ± 14.40 pmol/L	47.64 ± 12.34* pmol/L	0.487
			NR	52.83 ± 2.26		Group C (N=12): received 5 mg Glibenclamide per day		24.70 ± 1.43 kg/m^{2†}	25.54 ± 1.40 kg/m^{2†}	250.33 ± 14.55	154.50 ± 24.06*	11.86 ± 0.53	7.86 ± 0.45*	59.98 ± 17.83 pmol/L	59.68 ± 14.05* pmol/L	
**Viswanathan *et al.*, 2016**	50	T2DM subjects with more than 10 years duration and aged between 40 and 70 years, who had symptoms of peripheral neuropathic pain			Randomized controlled trial. The patients were randomly assigned to two groups: group 1 treated with the polyherbal formulation pain cream and group 2 or control group patients treated with a placebo cream.	Group 1 (N=26): patients were treated with the polyherbal formulation pain cream (Glycyrrhiza glabra 0.20%, Musa paradisiaca 19.42%, Curcuma longa 2.43%, Pandanus odoratissimus 9.70%, aloe vera 4.85% and coconut milk)	168 days	NR	NR	NR	NR	HbA1c values were significantly different through the baseline, until the final follow-up (p<0.005)		NR	NR	0.285
						Group 2 (N=24): patients were treated with a placebo cream		NR	NR	NR	NR	The mean HbA1c values were not significantly different through the baseline		NR	NR	
**Carvalho *et al.*, 2020**	21	T2DM subjects with oral diabetic drugs, age 18-80 years, Hb1Ac 7-10%. Pregnancy and patients with T2D complications were excluded	7 (33.4)	57.9 ± 9.22	Randomized double-blind and placebo-controlled pilot study. All participants were randomized in two groups: placebo and intervention	Placebo group (N=11) received two capsules per day of 600 mg/each, with microcrystalline cellulose (placebo).	30 days	70.5 (59.2 - 95.7)	69.8 (57.8 - 97.1)	223.2 (135 - 369)	226.8 (136.8 - 363.6)	NR	NR	NR	NR	0.832
						Intervention group (N=10): received two capsules per day of 600 mg/each, with ginger.		75.1 (46 - 90.3)	74.4 (46.3 - 89.5) *	198 (117 -318.6)	223.2 (111.6 - 261)	NR	NR	NR	NR	
**Fouad *et al.*, 2016**	72	T2DM Women. Insulin injection, lipid lowering medications, thyroid dysfunction, allergy to soya, family	0 (0)	46.53± 1.70	Intervention study with 2-arm. The study has two phases: phase 1 following low caloric balanced diet +	Group A (N=38): At phase 1, followed a low caloric balanced diet + soya bean flour mixed with 5% turmeric powder.	56 days (28 days at phase 1 and 28	93.03 ± 3.11	89.03 ± 3.03*	112.87 ± 4.66	99.53 ± 3.89 *	5.95 ± 0.14	4.93 ± 0.09	NR	NR	0.380

		history of cancer breast, pregnant or breast-feeding females were excluded			supplementation and phase 2 only following a low caloric balanced diet. Patients were divided in two groups (A and B)	Group B (N=34): low caloric balanced diet + bread formula with soya bean flour mixed with 5% turmeric powder.	days at phase 2)	87.24 ± 1.70	84.44 ± 1.72*	119.58 ± 5.48	103.19 ± 4.77*	6.17 ± 0.1	4.87 ± 0.08	NR	NR	
**Majeed *et al.*, 2021**	69	Prediabetic and newly diagnosed T2DM, aged 30-65 years. Prediabetes was defined as HbA1c 5.7–6.4% and FBS between 100 mg/ dL to 125 mg/dL. T2DM was defined as HbA1c value of 6.5–7.5% and FBS > 125 mg/dL. Pregnant and lactating women, patients with a history of acute or chronic illness and T1DM were excluded	9	49.6	Prospective, randomized, double-blind, active-controlled clinical trial. Enrolled patients were initially segregated into prediabetic patients and newly diagnosed diabetic patients. All the enrolled patients were randomized to two treatment groups: Treatment 1: GlycaCare-II as active or Treatment 2: Metformin as the comparator.	Prediabetic subject treatment I (N=17): received GlycaCare-II (522.5 mg), which containing cinnamon, bitter melon, Indian kino, Periploca of the Woods, Salacia, jambul and a piper nigrum	120 days	No significant differences in body weigh at the beginning and the end of the study in each of four groups		Significant decrease in FBS at the beginning and the end of the study in each of four groups		Significant decrease in Hb1Ac at the beginning and the end of the study in each of four groups		NR	NR	0.643
			3	48		Prediabetic subjects' treatment II (N=12): received metformin (500)								NR	NR	
			17	51.3		T2D subject treatment I (N=24): received GlycaCare-II (522.5 mg)								NR	NR	
			14	52.9		T2D subjects' treatment II (N=16): received metformin (500 mg)								NR	NR	
**Nganou-Gnindjio *et al.*, 2022**	21	T2DM subjects without changes in drugs therapy, aged above 21 years, with Hb1Ac 42-64 mmol/L. and chronic patients were excluded.	NR	54 [51-61]	Single-arm clinical trial. Data were collected during three visits: baseline, 21-day and 42-day follow-up.	Each participant received a capsule containing 399 mg of pure Zingiber officinale extracts three times per day for six weeks	42 days	31.1 [26.5-37.6] kg/m^{2†}	30 [26.5-37.6] kg/m^{2†}	6.6 [5.5-8.5] mmol/l	7.5 [6.9-8] mmol/l	49.7 [47.0-57.4] mmol/mol	44.3 [38.8-53] * mmol/mol	NR	NR	0.587
**Yu *et al.*, 2018**	450	Newly T2DM not-insulin diagnosed subjects, with Hb1Ac>7%, TG>1.7 mmol/L and TG< 5.65 mmol/L, age 18-70 years. Patients with lipid-lowering drugs and diabetic complications were excluded	104	52.82 ± 9.01	Multicentre randomized, positive-controlled, open-label clinical trial. All participants were randomized in two groups: JTTZ and metformin group. 225 subjects started in each group. Although only 215 and 199 participants in each group completed the study	JTTZ group (N=215): received a supplementation with 8 herbs (aloe vera, coptis chinensis, rhizoma anemarrhenae, red yeast rice, momordica charantia, salvia miltiorrhiza, schisandra chinensis and ginger)	84 days	77.82 ± 12.08	75.34 ± 12.05	9.64 ± 2.59 mmol/L	8.24 ± 2.35 mmol/L	8.26 ± 1.35	7.51 ± 1.44 *	103.89 ± 97.73 pmol/L	NR	0.642
			98	52.90 ± 8.52		Metformin group (N=199)		76.86 ± 12.06	74.83 ± 12.00	9.56 ± 2.39 mmol/L	8.21 ± 2.23 mmol/L	8.28 ± 1.33	7.57 ± 1.42 *	100.88 ± 131.62 pmol/L	NR	
**Blenvis *et al.*, 2007**	57	T2DM not-insulin dependent. Patients with Hb1Ac<6% and chronic disease were excluded	28 (49.1)	63.6	Randomized clinical trial. Enrolled subjects were stratified by sex and randomized to receive either cinnamon	Placebo group (N=28): received two capsules per day. Each capsule contained 500 mg of placebo.	84 days	32.5 ± 1.7	NS	132.9 ± 9.3	NS	7.2 ± 0.3	NS	12.9 ± 1.3	NS	0.404
				58.0		Cinnamon group (N=29): received two capsules per		32.0 ± 1.5	NS	144.7 ± 10.4	NS	7.1 ± 0.2	NS	11.8 ± 1.6	NS	

					(C. cassia) or placebo (wheat flour).	day. Each capsule contained 500 mg of cinnamon (1000 mg of cinnamon per day)										
**Mirmiran *et al.*, 2019**	41	Newly diagnosed T2DM subjects, FBS>180 and <250 mg/dL, BMI 18.5- 30 kg/m^2, without insulin therapy. Patients with chronic disease were excluded.	8 (40%)	58.90 ± 7.93	Randomized double-blind placebo-controlled trial. The selected patients were randomly divided into two groups: cinnamon or placebo group	Cinnamon group (N=19): received three capsules Daily (3 g cinnamon/day)	56 days	73.75 ± 10.74	NR	No significant difference between pre and post intervention		No significant difference between pre and post intervention		No significant difference between pre and post intervention		0.803
			7 (36.8)	56.26 ± 9.46		Placebo group (N=22): received three capsules of microcrystalline cellulose as placebo daily		77.15 ± 15.63	NR	No significant difference between pre and post intervention		No significant difference between pre and post intervention		No significant difference between pre and post intervention		
**Mirmiranpour *et al.*, 2020**	136	T2DM not-insulin therapy subjects, age 40-60 years, FBS: 125-250 mg/dL and Hb1Ac 7-8%. Pregnancy, consumption of specific medicines or chronic disease were excluded.	13	58.4 ± 11.4	Randomized placebo-controlled and 4 arm trial. All participants were randomly divided into four groups matched for age and gender: A, B, C or D group	Group A (N=30): received a capsule of Lactobacillus acidophilus 10^8 cfu and 0.5 g/day of cinnamon.	84 days	79.5 ± 11.8	NR	NR	147.7± 3.71**	NR	7.66 ± 1.11**	NR	NR	0.660
			10	59.7 ± 12.2		Group B (N=30): received a capsule of Lactobacillus acidophilus 10^8 cfu		79.3 ± 13.3	NR	NR	150.43 ±43.36 **	NR	7.42 ± 1.23**	NR	NR	
			14	58.8 ± 12.8		Group C (N=28): received 0.5 g/day of cinnamon		81.6 ± 12.6	NR	NR	152.5± 48.1**	NR	7.68 ± 0.83**	NR	NR	
			12	58.2 ± 11.8		Group D (N=27): received a capsule (0.5 g/day) of rice flour		83.5 ± 13.6	NR	NR	177.3 ± 23.02	NR	8.48 ± 0.59	NR	NR	
**Tanzidi-Roodi *et al.*, 2023**	30	T2DM not-insulin therapy subjects, age 40-75 years, BMI 25-35 kg/m^2. Pregnancy, consumption of specific medicines or chronic disease were excluded	6 (40)	57.20 ±7.95	Randomized, triple-blind, placebo-controlled clinical trial. All patients were randomized in two groups: intervention or placebo	Intervention group (N=15): received 500 mg of Viabet® capsules which containing fenugreek, nettle, cinnamon, stevia and aconite bean	84 days	83.30 ±9.20	81.7 ± 8.98*	164.2 ±55.63	162.8 ±55.31	7.59 ± 1.69	6.40 ± 1.45*	NR	NR	0.821
			4 (26.7)	55.20 ±6.26		Placebo group (N=15): received placebo capsule		75.60 ±7.98	75.7 ± 8.17	132.6 ± 75.04	144 ± 81.79	6.35 ± 1.37	7.26 ± 1.43	NR	NR	
**Wainstein *et al.*, 2011**	59	T2DM not-insulin therapy subjects, older 30 years, Hb1Ac 6.5 – 10.5%. Pregnancy, patients with known sensitivity to cinnamon or chronic disease were excluded	14 (48.3)	61.7 – 6.3	Single-center randomized, placebo-controlled clinical trial. All participants were randomized in two groups: cinnamon or placebo	Cinnamon group (N=29): received 6 capsules per day with 400 mg of cinnamon each capsule (2.4 g cinnamon/day).	84 days	85.1 ± 15.2	-0.03 ± 2.2[1]	165.0 ± 35.9	13.4 ± 38.9[1]	8.0 ± 1.2	0.61 ± 0.87[1]	15.5 ± 10.2	0.86 ± 8.03[1]	0.821
			21 (60)	64.4 – 15.4		Placebo group (N=30): received 6 capsules per day of placebo capsules, which contained 400 mg of microcrystalline cellulose.		88.7 ± 20.2	- 0.22 ± 2.8[1]	170.1 ± 46.3	11.8 ± 47.9[1]	8.1 ± 1.1	0.30 ± 0.98[1]	16.2 ± 16.7	16.2 ± 16.7[1]	
**Zare *et al.*, 2018**	138	T2DM not-insulin dependent subjects, age 30-80 years, BMI 18-40 kg/m^2, FBS 126-250 mg/dL	32 (46.3)	52.1 ± 9.7	Randomized triple-blinded, placebo-controlled clinical trial. All patients were randomly assigned in four groups: cinnamon (BMI > 27, BMI < 27) and Placebo (BMI > 27, BMI < 27).	Cinnamon group BMI>27 (N=36): received 1000 mg of cinnamon daily	84 days	75.7 ± 11.6	-1.90 ±0.26 **	-19.37 ± 2.3**		-0.42 ± 0.06**		-2.38 ± 0.30** mIU/L		0.933
						Cinnamon group BMI<27 (N=33): received 1000 mg of cinnamon daily				-5.8 ± 1.62		-0.93 ± 0.02		-1.07 ± 0.24** mIU/L		
			43 (62.3)	53.2 ± 8.5		Placebo group BMI>27 (N=37): received 1000 mg of placebo daily		74.4 ± 11.6	0.19 ± 0.15	-0.22 ± 1.53		0.044 ± 0.01		0.003 ± 0.02 mIU/L		

						Cinnamon group BMI<27 (N=32): received 1000 mg of placebo daily				-3.3 ± 1.02	-0.048 ± 0.03	0.008 ± 0.03 mIU/L	

*denotes a significant difference after intervention or supplementation; **denotes a significant decrease compared to the control group; 1indicated change from baseline.
† indicated BMI, due to body weight was not reported. NR: Not reported.. PA: Physical activity. HIIT: High intensity interval training. HD: hemodialysis; cfu: Colony forming units; T2DM: Type 2 diabetes mellitus; T1DM: Type 1 diabetes mellitus; CVD; Cardiovascular disease; FBS: Fasting blood sugar; TG: Triglycerides, LDL-c; low density lipoprotein cholesterol; eGFR: estimated glomerular filtration rate.

Supplemental Table 3. Evaluation of Quality Assessment instruments for randomized controlled trials included in the systematic study.

Instruments	Objective sufficiently described	Study design	Method of subject	Comparison group	Random allocation	Blinding of investigators	Blinding of subjects	Outcome and exposure measure(s)	Sample size	Analytic methods	Estimate of variance	Controlling for confounding	Results reported	Conclusion supported
**Moghadam *et al.*, 2021**	●	●	●	●	●	○	●	●	◐	◐	●	◐	◐	◐
**Blevins *et al.*, 2007**	○	◐	○	◐	◐	◐	◐	○	○	◐	◐	○	◐	●
**Mirmiran *et al.*, 2019**	●	●	●	●	●	○	●	●	●	◐	●	○	◐	◐
**Mirmiranpour *et al.*, 2020**	●	●	●	●	●	○	○	●	●	◐	●	○	◐	◐
**Tanzidi *et al.*, 2023**	●	●	●	●	●	○	●	●	●	◐	●	◐	●	◐
**Wainstein *et al.*, 2011**	●	●	◐	●	○	○	●	●	●	◐	●	◐	◐	◐
**Zare *et al.*, 2018**	●	●	●	●	●	●	●	●	●	◐	●	◐	●	●
**Ali *et al.*, 2021**	◐	◐	◐	◐	○	○	○	◐	○	◐	●	○	●	○
**Assaad-Khalil *et al.*, 2022**	●	●	●	●	●	●	●	●	●	◐	●	◐	◐	◐
**Bamosa *et al.*, 2010**	●	●	●	●	○	○	○	○	○	◐	●	◐	◐	◐
**Bilal *et al.*, 2019**	◐	●	◐	○	○	NA	NA	◐	●	◐	◐	○	◐	◐
**El-Shamy *et al.*, 2011**	●	●	◐	●	◐	○	○	◐	○	◐	●	●	●	◐

**Moustafa *et al.*,2019**	●	●	●	●	●	○	○	●	○	◐	●	◐	●	◐
**Rao *et al.*, 2020**	●	◐	◐	●	NA	NA	NA	●	●	◐	●	◐	◐	◐
**Zarvandi *et al.*, 2017**	●	◐	○	○	○	○	○	◐	○	◐	●	○	◐	○
**Banerji *et al.*, 2016**	◐	○	○	○	NA	NA	NA	◐	○	◐	●	○	●	○
**Faiza *et al.*,l 2009**	●	●	●	●	●	●	●	●	●	○	●	○	●	●
**Ferreira de Sousa *et al.*, 2020**	●	●	●	●	◐	◐	◐	●	●	◐	●	◐	●	◐
**Iyer *et al.*, 2010**	●	●	●	○	○	○	○	●	○	○	●	○	◐	◐
**Kurian *et al.*, 2014**	◐	●	●	●	NA	NA	NA	◐	○	◐	◐	●	●	◐
**Mahajan *et al.*, 2015**	◐	◐	◐	◐	NA	NA	NA	◐	○	●	●	○	●	◐
**Mani *et al.*, 1997**	◐	◐	◐	◐	○	○	○	●	○	◐	◐	○	◐	○
**Singh *et al.*, 2016**	●	◐	○	◐	●	○	○	◐	○	○	◐	○	●	◐
**Sukandar *et al.*,2014**	●	◐	◐	◐	○	○	○	◐	○	◐	●	○	◐	◐
**Sukandar *et al.*, 2010**	●	●	●	◐	○	○	○	◐	○	◐	●	◐	◐	◐
**Viswanathan *et al.*, 2016**	●	◐	●	●	○	○	○	●	○	○	○	○	○	◐
**Carvalho *et al.*, 2020**	●	●	●	●	●	●	●	●	○	●	◐	◐	●	●

**Foaud *et al.*, 2016**	●	◐	◐	◐	○	○	○	◐	○	●	◐	○	◐	◐
**Majeed *et al.*, 2021**	●	●	●	●	◐	◐	◐	●	○	◐	◐	◐	◐	◐
**Nganou-Gnindijo *et al.*, 2022**	●	◐	◐	●	NA	NA	NA	●	●	◐	●	◐	◐	◐
**Yu *et al.*, 2018**	●	●	●	●	●	○	○	●	○	◐	●	◐	●	◐
**Quirarte-baez *et al.*, 2019**	◐	◐	○	○	○	○	○	◐	○	○	●	○	◐	○

* Symbology significance and scoring is as follows: ● Yes (2 points); ◐ Partial (1 point); ○ No (0 point); NA denotes "Not applicable"; Complete description of the issues included in que quality assessment are: 1) Question, objective sufficiently described?; 2) Study design evident and appropriate?; 3) Method of subject, comparison group selection or source of information, input variables described and appropriate?; 4) Subject and comparison group (if applicable) characteristics sufficiently described?; 5) If interventional and random allocation was possible, was it reported?; 6)If interventional and blinding of investigators was possible, was it reported?; 7) If interventional and blinding of subjects was possible, was it reported?; 8) Outcome and (if applicable) exposure measure(s) well defined and robust to measurement, misclassification bias? Means of assessment reported?; 9) Sample size appropriate?; 10) Analytic methods described, justified and appropriate?; 11) Some estimate of variance is reported for the main results?; 12) Controlling for confounding?; 13) Results reported in sufficient detail?; 14) Conclusion supported by the results?
