# Supplementary figures and images for: Effect of Aromatic Herbs and Spices Present in the Mediterranean Diet on the Glycemic Profile in Type 2 Diabetes Subjects: A Systematic Review and Meta-Analysis
Source: Nutrients. 2024 Mar 7;16(6):756. doi: 10.3390/nu16060756 (PMC10975382; doi:10.3390/nu16060756)

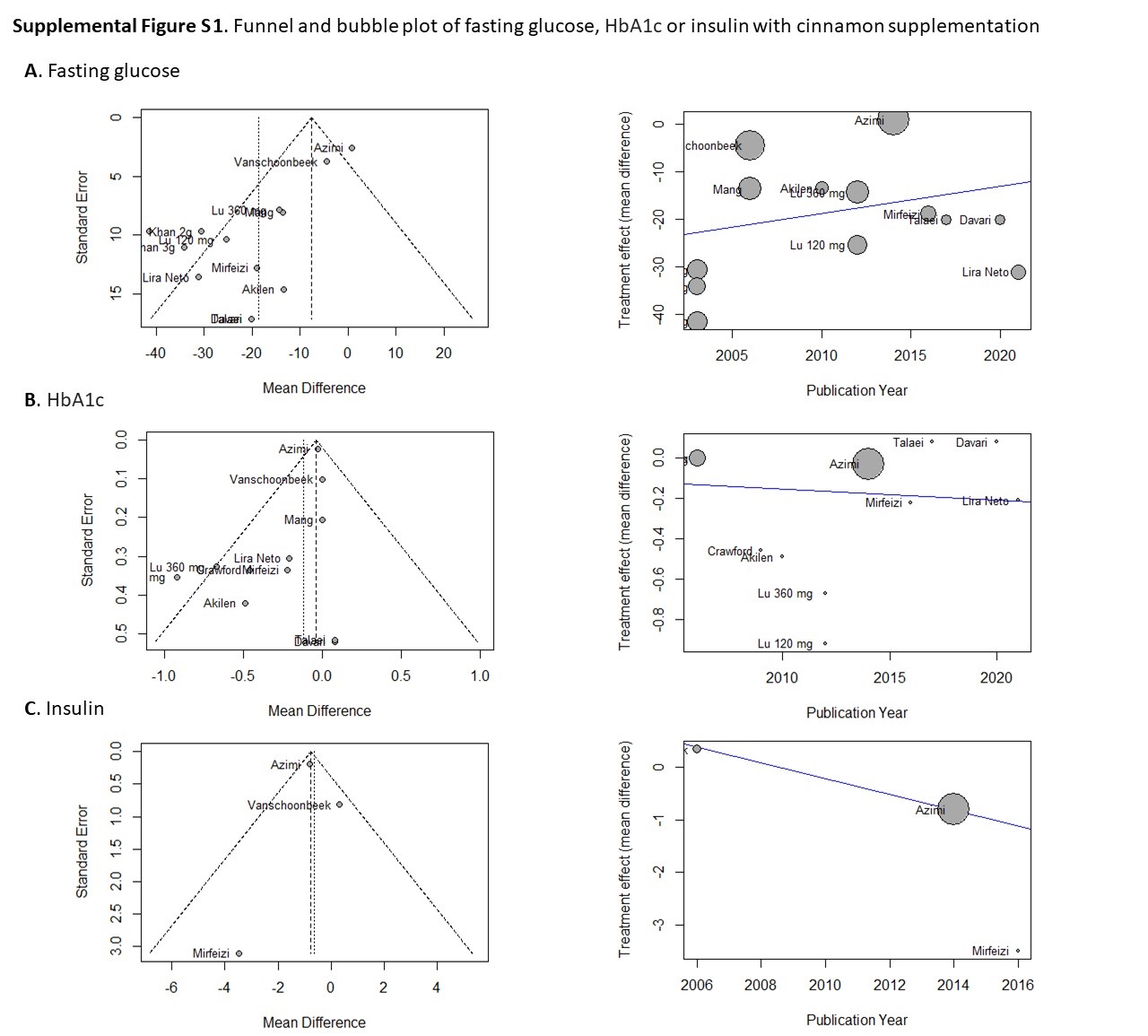

Supplement: Supplementary file 1 [file nutrients-16-00756-s001.zip › Supplemental figure S1.JPG]

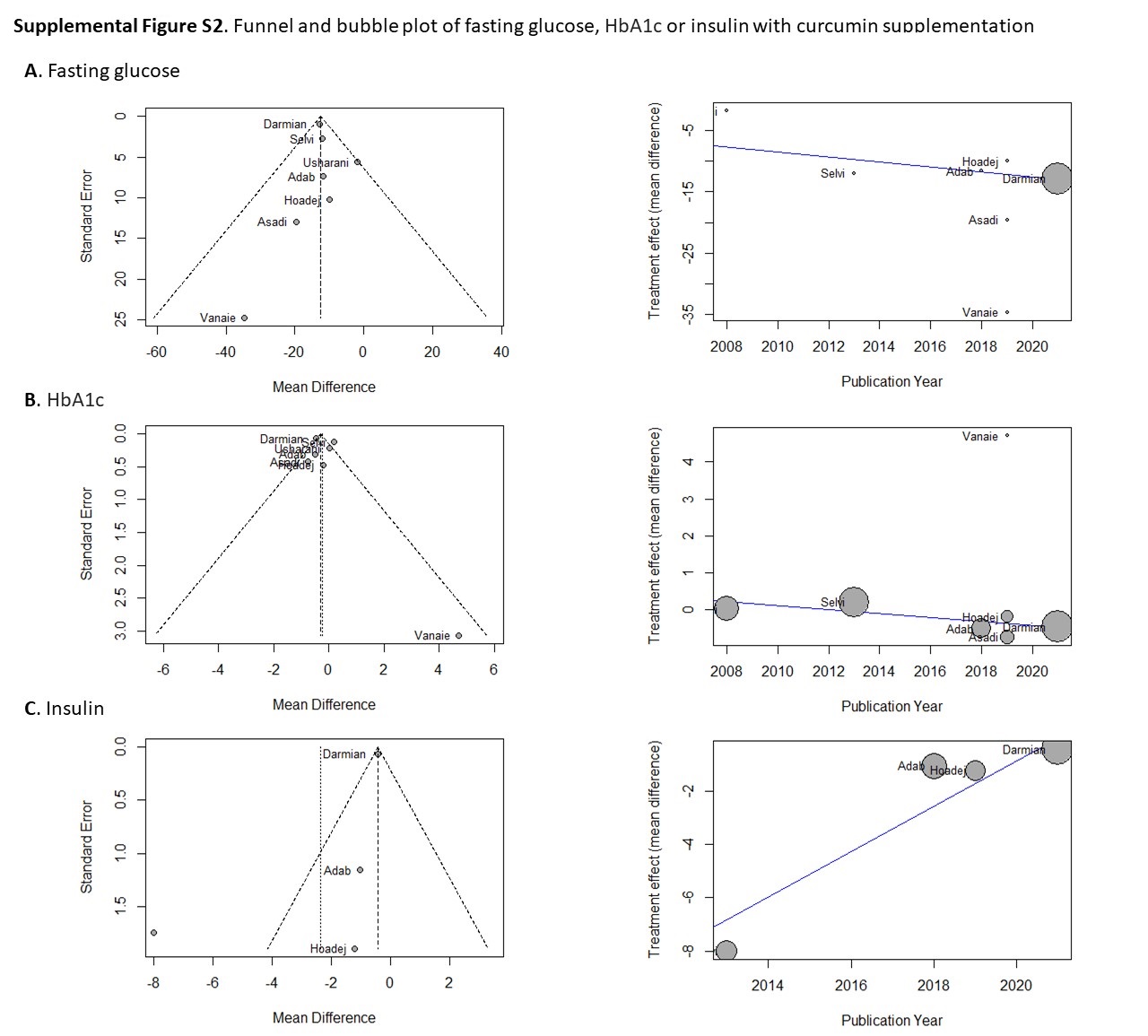

Supplement: Supplementary file 1 [file nutrients-16-00756-s001.zip › Supplemental figure S2.JPG]

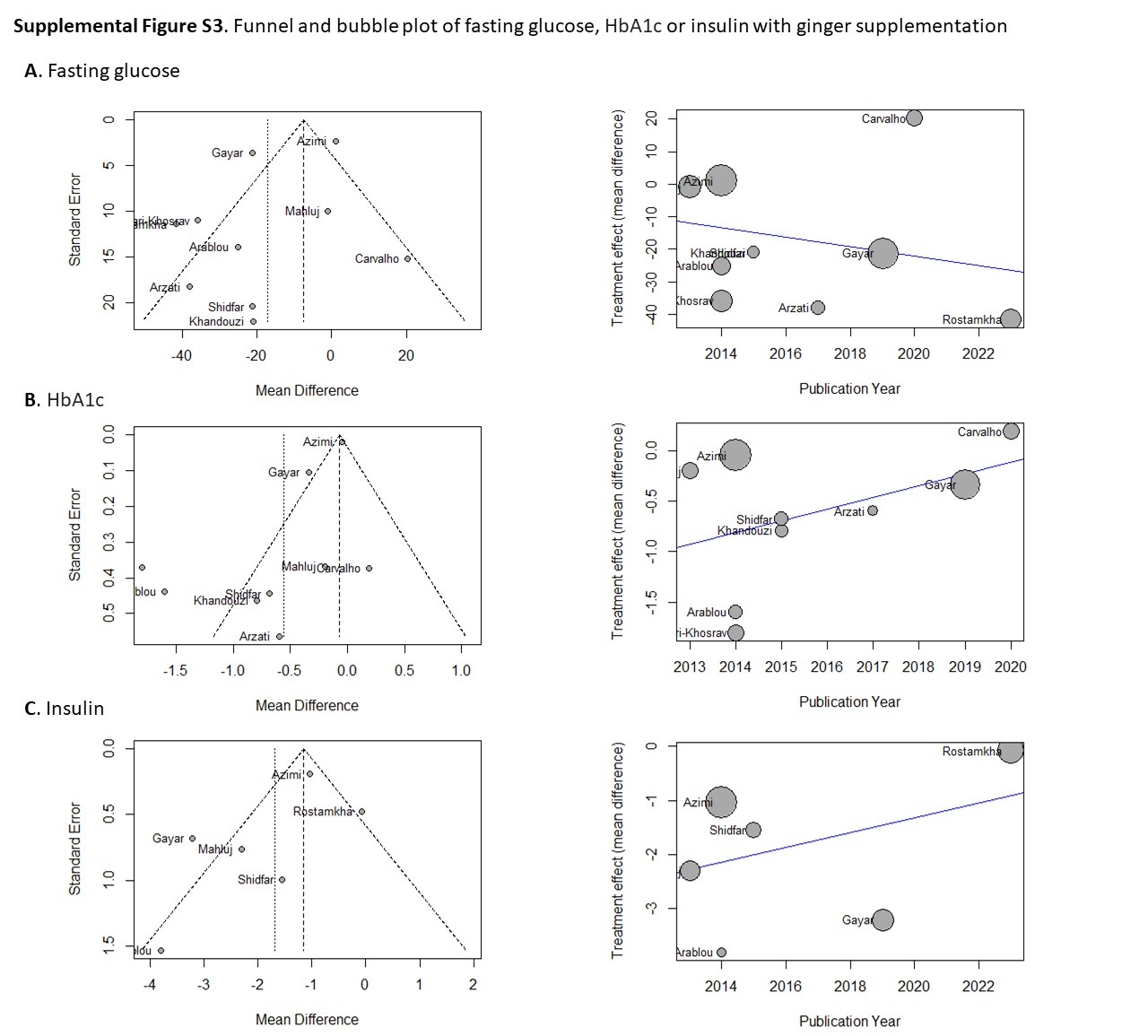

Supplement: Supplementary file 1 [file nutrients-16-00756-s001.zip › Supplemental figure S3.JPG]

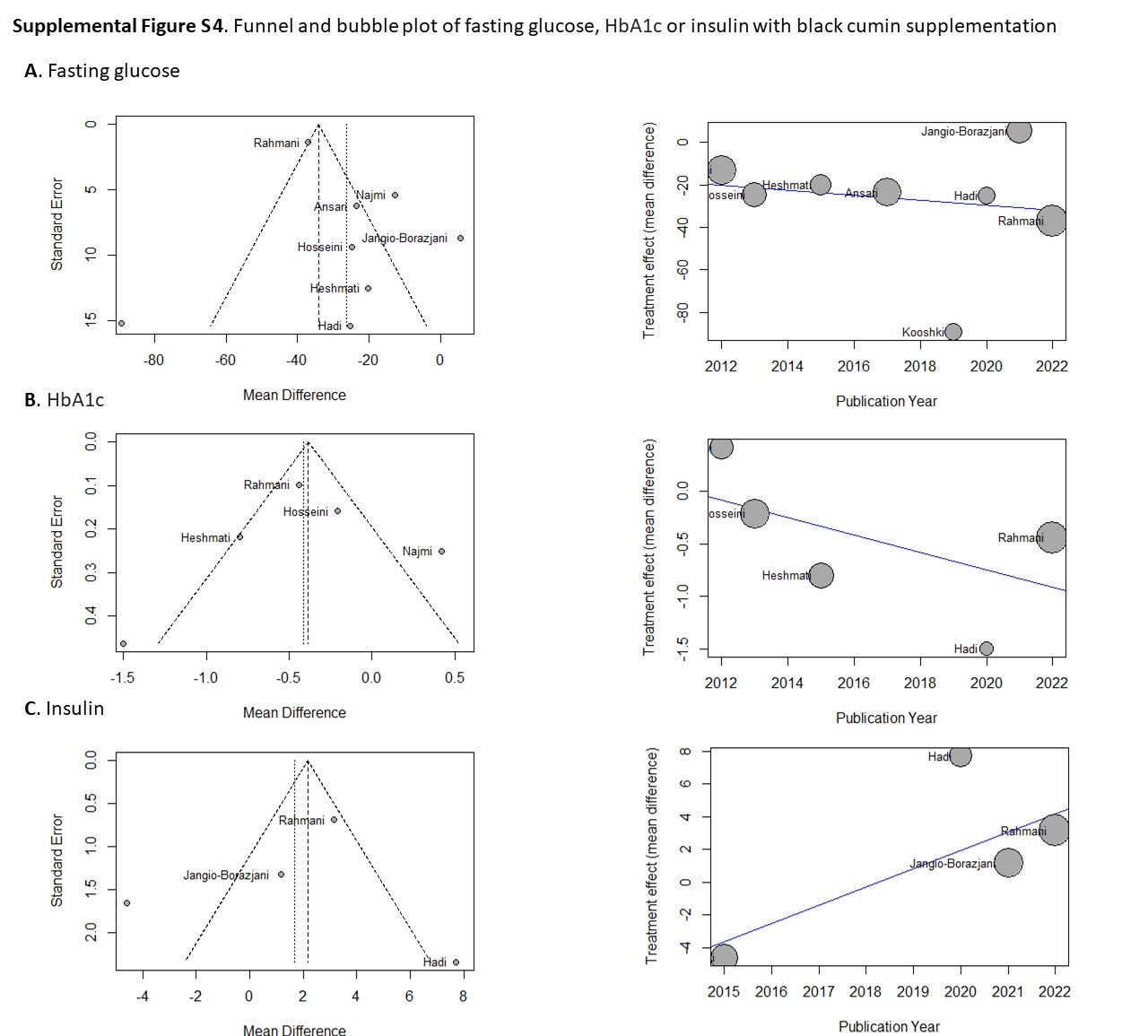

Supplement: Supplementary file 1 [file nutrients-16-00756-s001.zip › Supplemental figure S4.JPG]

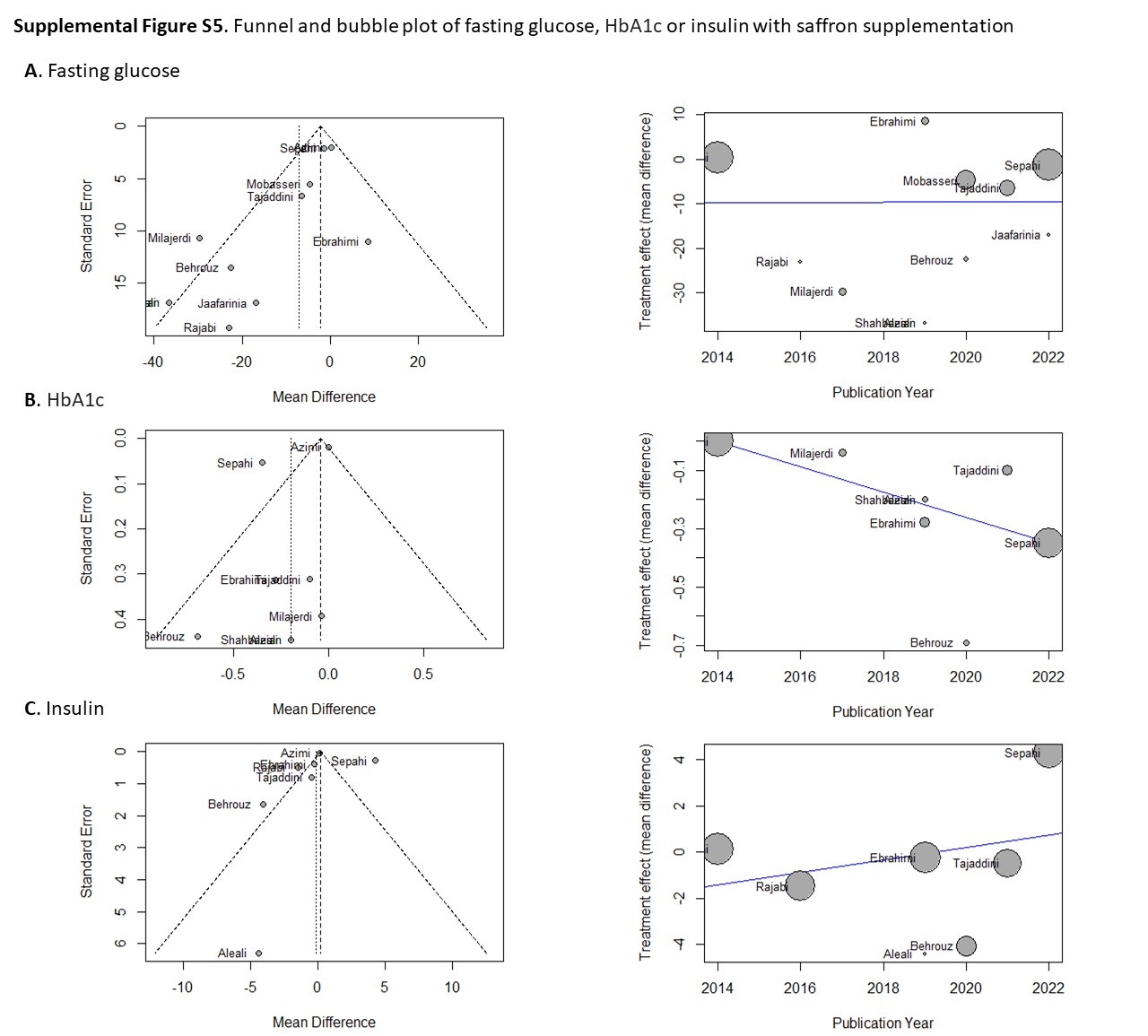

Supplement: Supplementary file 1 [file nutrients-16-00756-s001.zip › Supplemental figure S5.JPG]
